# pH-Responsive Sodium Alginate/Carboxymethyl Cellulose Hydrogels for Enhanced Stability and Gastrointestinal Sustained Release Delivery of Chlorogenic Acid

**DOI:** 10.3390/polym18091087

**Published:** 2026-04-29

**Authors:** Lanxin Ke, Linqing Qian, Yincong Chen, Yanchen Ren, Meiqi Shi, Kun Wang, Ting Wang

**Affiliations:** 1 College of Chemistry, Chemical Engineering and Resource Utilization, Northeast Forestry University, Harbin 150040, China; kelanxin@nefu.edu.cn (L.K.); qianlinqing@nefu.edu.cn (L.Q.); 2023223340@nefu.edu.cn (Y.C.); renyanchen@nefu.edu.cn (Y.R.); shimeiqi@nefu.edu.cn (M.S.); 2 Harbin Vocational College of Science and Technology, Harbin 150040, China

**Keywords:** chlorogenic acid, alginate hydrogel, carboxymethyl cellulose, pH-responsive release, gastrointestinal delivery

## Abstract

Chlorogenic acid (CGA) is a natural polyphenol with various biological activities, but its poor stability and premature release in the gastrointestinal tract limit oral application. Herein, a pH-responsive bilayer hydrogel based on sodium alginate (SA) and carboxymethyl cellulose (CMC) was developed to enhance the gastrointestinal stability and controlled release of CGA. CGA-loaded SA hydrogels were prepared via Ca^2+^-induced ionotropic gelation, followed by CMC coating to form a bilayer structure. The SA/CMC hydrogels showed a drug loading capacity of 15.2–16.7% and pH-dependent swelling behavior. In vitro release studies revealed that the bilayer hydrogel suppressed CGA release in simulated gastric fluid (pH 1.2), with a cumulative release of approximately 30%, while enabling sustained release in simulated intestinal fluid (pH 6.8), reaching about 70% within 10 h. Release kinetics indicated that CGA release was controlled by Fickian diffusion under acidic conditions and by a diffusion-polymer relaxation mechanism under intestinal conditions. Moreover, encapsulation in the SA/CMC hydrogel improved the thermal, light, and pH stability of CGA while maintaining its antioxidant activity and biocompatibility. These results indicate that SA/CMC bilayer hydrogels provide a promising strategy for stabilized gastrointestinal delivery of chlorogenic acid.

## 1. Introduction

In recent years, the use of bioactive compounds that combine natural properties, safety, and multiple health benefits has continued to grow in the food, pharmaceutical, and packaging industries [1,2]. Among these, chlorogenic acid (CGA), an important class of natural bioactive polyphenolic compounds, is widely found in natural foods such as fruits, vegetables, coffee beans, and tea leaves [3,4,5]. Extensive research has demonstrated that CGA possesses significant antioxidant, anti-inflammatory, antibacterial, antitumor, antidiabetic, and neuroprotective effects [6,7,8], and shows great potential for application in the treatment of gastrointestinal diseases [9,10]. Given its high biological activity and utility, CGA has garnered attention and found applications across multiple sectors: in the pharmaceutical sector, it serves as a functional active ingredient in the development of novel drugs and drug delivery systems [11,12,13,14]; in the livestock sector, its addition to animal feed helps regulate the gut microbiota and improve reproductive physiological indicators [15,16]; and in the consumer goods sector, CGA is widely used in the formulation of cosmetics and household chemicals due to its antioxidant and antibacterial properties [17,18].

However, CGA still faces multiple critical challenges in practical research and application, primarily related to insufficient structural stability, low bioavailability, and application safety [8]. From a molecular structural perspective, CGA contains ester bonds, unsaturated double bonds, and unstable polyphenolic groups, making it prone to intramolecular ester migration and isomerization reactions. It also decomposes under thermal, light, and oxidative conditions, thereby limiting its stability during processing, storage, and use [19]. Furthermore, CGA has a short shelf life and interacts with various components in the body, potentially causing adverse effects, which further limits its large-scale application. Regarding in vivo behavior, CGA also faces issues such as low intestinal absorption efficiency, limited bioavailability following oral administration, complex metabolic pathways, and significant interindividual variability [20,21,22,23]. These factors collectively make it difficult to achieve effective protection and precise release in the gastrointestinal environment, constituting the core bottleneck constraining the oral application and functional efficacy of chlorogenic acid.

To address the issues of poor stability and low bioavailability of CGA, previous studies have attempted to improve its in vivo and in vitro behavior by developing delivery systems such as emulsions and liposomes, and have made some progress [24,25]. However, the application of such systems in the complex gastrointestinal environment has obvious limitations. Emulsion systems are prone to structural disruption under gastrointestinal conditions, exhibit limited environmental stability, and their release behavior is difficult to precisely control, resulting in relatively restricted application scenarios [26]. Liposomes, although capable of encapsulating bioactive molecules, frequently suffer from insufficient mechanical stability, drug leakage, and limited storage stability, which may restrict their practical applications in oral delivery [27].

In contrast, hydrogel delivery systems based on natural polymers combine excellent structural stability, biocompatibility, and controllable release properties, and are considered ideal candidates for the gastrointestinal delivery of active substances [28,29]. Among the commonly used biopolymers, sodium alginate (SA) and carboxymethyl cellulose (CMC) have received considerable attention due to their natural origin, low toxicity, and strong gel-forming ability. Previous studies have demonstrated that alginate-based hydrogels formed through calcium ion cross-linking possess favorable encapsulation capability and structural stability, while the introduction of CMC can further enhance the mechanical properties and modulate swelling behavior through intermolecular interactions [30,31,32]. Such composite hydrogel systems provide an effective platform for constructing pH-responsive carriers capable of regulating the release behavior of encapsulated compounds.

To the best of the authors’ knowledge, there are currently no reports specifically describing the use of sodium alginate–carboxymethyl cellulose (SA-CMC) composite hydrogels for chlorogenic acid (CGA) delivery. Moreover, systematic investigations into the potential of such systems to enhance the gastrointestinal stability and oral bioavailability of natural polyphenolic compounds remain limited. In the field of polyphenol delivery, several related studies provide partial insights. For instance, one study employed reverse vesicularization (RVS) to fabricate liquid-core SA hydrogel beads for CGA encapsulation, primarily focusing on microstructural mechanical properties and release kinetics, while the impact on CGA bioactivity was not addressed [33]. Another study developed SA/CMC-based gel systems incorporating rose anthocyanins to produce colorimetric films for shrimp freshness monitoring; however, the functional stability and activity of the encapsulated anthocyanins were not systematically evaluated [34]. In addition, a gelatin/carboxymethyl cellulose/peach gum ternary microcapsule system was used to encapsulate sweet orange essential oil, where phenolic-rich peach gum functioned as a structural component and exhibited antioxidant activity, suggesting potential applications in food preservation [35]. Similarly, eucalyptus polyphenols have been incorporated into lyophilized SA/CMC matrices, with reported antioxidant and antibacterial properties, although stability performance and pH-responsive release behavior were not investigated [36]. Furthermore, studies utilizing sodium alginate or cellulose-based matrices for procyanidin encapsulation have evaluated stability and antioxidant activity, partially supporting the feasibility of polysaccharide-based systems for polyphenol delivery [37,38].

Based on the design concept of “structural compartmentalization regulation function,” this study employed the Ca^2+^ cross-linking method to construct a chlorogenic acid-loaded SA-CMC hydrogel delivery system. The effectiveness of this system in enhancing chlorogenic acid stability was evaluated, and its ability to retain chlorogenic acid bioactivity was validated through antioxidant performance tests and cytotoxicity experiments. These findings provide a feasible structural design strategy for gastrointestinal stabilization and delivery of unstable natural polyphenolic bioactive compounds.

## 2. Materials and Methods

### 2.1. Materials

Carboxymethyl cellulose (DS = 1.2), sodium alginate (viscosity 200 ± 20 mPa·s), 2,2-diphenyl-1-picrylhydrazyl (purity 97%), thiazolyl blue tetrazolium bromide (MTT) and chlorogenic acid (purity 95%) were all purchased from Aladdin Reagent (Shanghai) Co., Ltd. (Shanghai, China). Sodium chloride, anhydrous sodium dihydrogen phosphate, disodium hydrogen phosphate dodecahydrate, DMSO (purity 99.7%) and anhydrous calcium chloride were all purchased from Rhawn Reagent (Shanghai) Co., Ltd. (Shanghai, China). Other reagents used were commercially available and of analytical grade. All the above materials were from designated sources and used without any purification.

### 2.2. Preparation of CGA-SA-CMC Gel Hydrogels

**Figure 1:**
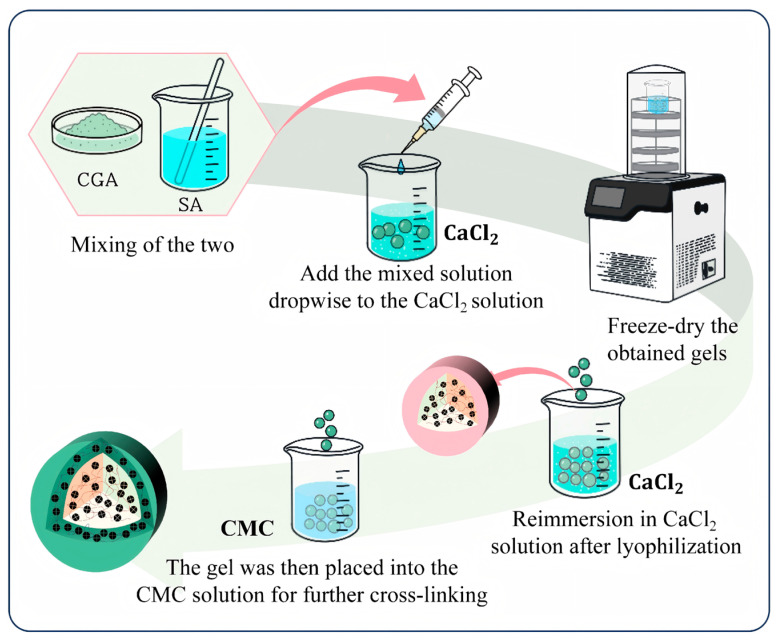
Preparation of CGA-SA-CMC gel hydrogels.

The preparation process of CGA-SA-CMC Gel hydrogels is illustrated in Figure 1. A certain amount of SA was dissolved in deionized water to prepare a 1.6 wt% SA solution, and chlorogenic acid (CGA) accounting for one quarter of the mass of SA was added. The CGA:SA mass ratio was determined based on a preliminary screening study. A series of formulations with CGA:SA mass ratios of 1:5, 1:4, 1:3, 1:2, and 1:1 were prepared to evaluate drug loading capacity. At a 1:5 ratio, although hydrogel formation was achieved, the CGA content was insufficient for reliable UV–Vis spectrophotometric quantification, preventing accurate calculation of drug loading. In contrast, ratios ranging from 1:4 to 1:1 enabled successful CGA encapsulation and reproducible measurements. Among these, the 1:4 ratio was selected as a representative and balanced formulation for subsequent experiments due to its stable encapsulation behavior and reliable analytical performance, and the reported drug loading capacity corresponds to this specific ratio and does not represent the maximum loading capacity of the system. The mixture was stirred to dissolve until the solution was uniform and transparent, then the solution was dropped uniformly into a 3 wt% calcium chloride solution with a syringe to obtain CGA-SA microspheres, which were then stirred and incubated at room temperature for 40 min. The obtained hydrogels were freeze-dried, then immersed in calcium chloride solution for about 5 min. After absorbing a certain amount of the solution, they were quickly put into a 0.75 wt% CMC solution, stirred and incubated at room temperature for 20 min to obtain CGA-SA-CMC hydrogels. The hydrogels were then taken out, rinsed 2 to 3 times with deionized water and freeze-dried. The preparation methods of other control samples including SA, SA-CMC and CGA-SA were the same as above. All the preparation processes of CGA-loaded hydrogels were carried out at room temperature in the dark.

### 2.3. Characterization of Hydrogels

Fourier Transform Infrared Spectroscop: FTIR was used to analyze the components of CGA-SA, CGA-SA-CMC and SA-CMC samples. The freeze-dried samples were ground with a pestle in an agate mortar, and the ground samples were mixed with potassium bromide at a ratio of 1:100. Subsequently, the mixture was compressed into a 1 mm translucent disk by applying a pressure of 20 MPa for 30 s. An FTIR spectrometer (Nicolet IS10, Thermo Fisher Scientific, Waltham, MA, USA) was used to record the FTIR spectra in the wavelength range of 4000–500 cm^−1^.

X-ray diffraction: X-ray diffractometer (Rigaku D-MAX 2550, Tokyo, Japan) was employed to analyze and confirm the presence of crystal structures for CGA, SA-CMC, and CGA-SA-CMC. The experiment utilized Cu Kα1 target radiation source as the incident source, with a scanning rate of 15 min^−1^ and an angular range of 5° to 80° to measure the diffraction patterns of CGA, SA-CMC, and CGA-SA-CMC, yielding corresponding diffraction spectra.

Scanning Electron Microscopy: SEM (JSM-7500, JEOL, Tokyo, Japan) was used to detect the morphology of freeze-dried CGA-SA and CGA-SA-CMC hydrogels. The freeze-dried samples were fixed on aluminum stubs with double-sided adhesive tape. Then, the ultra-thin gold layer of the sputtered samples was directly observed with SEM at an accelerating voltage of 10 kV.

### 2.4. Properties of Hydrogels

#### 2.4.1. Study on Swelling Property

To study the equilibrium swelling behavior of the hydrogels, CGA-SA-CMC samples were placed in buffer solutions with pH = 6.8 and pH = 1.2 for swelling at 37 °C. The hydrogels were weighed at regular intervals, and the swelling was considered to reach equilibrium when the mass showed almost no change between two consecutive weighings.

#### 2.4.2. Determination of Total Drug Loading Capacity of Hydrogels

Accurately weigh 10 mg of dried drug-loaded hydrogels and place them in a beaker containing 100 mL of pH = 7.4 PBS solution. The beaker was placed in a constant temperature water bath at 15 °C and stirred rapidly in the dark for 24 h until the hydrogels were completely broken. The supernatant was collected by centrifugation, and the absorbance A at 330 nm was determined with an ultraviolet-visible spectrophotometer (UV-2700, Shimadzu, Kyoto, Japan). The concentration of the supernatant was measured according to the CGA standard working curve Formula (1), and the average value was taken from three parallel experiments. The total drug loading capacity Q_33_ of the sample was calculated by Formula (2) [39]. The CGA encapsulation efficiency was calculated using Formula (3).

(1) A=kc+b

(2) $$Q_{33}=\frac{c\times V}{m}\times 100\%$$

(3) $$EE=\frac{c\times V}{M}\times 100\%$$

where A is the absorbance at 330 nm; k and b are the slope and intercept of the standard working curve respectively; c is the concentration of CGA in the supernatant (mg/mL); V is the volume of PBS solution (mL); m is the mass of dried drug-loaded hydrogels (mg); M is the mass of total drug.

#### 2.4.3. Study on Chlorogenic Acid Release Behavior

Accurately weigh 10 mg of dried CGA-SA-CMC samples and place them in a beaker containing 100 mL of simulated gastric fluid with pH = 1.2. The beaker was placed in a constant temperature water bath at 37 °C with a certain oscillation speed. At an interval of 1 h, 3.00 mL of the supernatant was quickly taken out, and 3.00 mL of simulated gastric fluid with pH = 1.2 was added to the solution at the same time. This operation was maintained for sampling for 10 h. Finally, the absorbance A of the collected supernatant at 330 nm was measured with an ultraviolet-visible spectrophotometer, and the drug concentration c in the solution at different times was calculated by using the CGA standard curve obtained from previous experiments. The average value was calculated from three parallel experiments. In addition, the drug release behavior of each sample in simulated intestinal fluid with pH = 6.8 was determined by the same method. The cumulative release of chlorogenic acid was expressed by Formula (3) [40].

(4) $$Q_{cum}=\frac{V\times \sum_{i=1}^{n}\;\;C_{i}+V_{0}\times C_{n}}{M\times Q}\times 100\%$$

where V is the volume of the extract in the release system (3.00 mL); $V_{0}$ is the initial volume of the solution (100 mL); $C_{i}$ and $C_{i-1}$ are the concentrations of chlorogenic acid in the release medium at the i-th and (i − 1)-th time respectively; Q is the loading percentage of chlorogenic acid; M is the weight of the hydrogel (g).

#### 2.4.4. Study on Chlorogenic Acid Release Kinetics

The experimental method was similar to that of the release behavior study, with a sampling interval of 20 min. The drug release percentage of hydrogels is usually expressed by the Korsmeyer-Peppas kinetic model, which is a model describing that the drug release rate is proportional to the power function of the drug content index n with time. The drug release formula is shown in Formula (4) [41].

(5) $$\frac{M_{t}}{M_{\infty }}=kt^{n}$$

where $M_{t}$ is the drug release amount at time t (mg); $M_{\infty }$ is the maximum drug release amount at the end time (mg); k is the release constant of the Korsmeyer-Peppas model; n is the release index. Linear fitting was performed with lnt as the abscissa and $ln(M_{t}/M_{\infty })$ as the ordinate according to the experimental results.

### 2.5. Study on Chlorogenic Acid Stability

#### 2.5.1. Thermal Stability Test

Chlorogenic acid is a thermally unstable substance. Considering that CGA is often used in cosmetics, medicine, health products and other fields and usually undergoes heating or high-temperature treatment, room temperature (25 °C), digestive temperature (37 °C) and high-temperature sterilization temperature (65 °C, general sterilization time is 20 min) were selected to compare the stability of chlorogenic acid solution and CGA-SA-CMC in heating environment. That is, the effects of three different heat treatment conditions on the stability of chlorogenic acid hydrogels were studied, which were long-term heating at room temperature (25 °C, 24 h), long-term heating at body temperature (37 °C, 4 h) and short-term heating at high temperature (65 °C, 20 min). The concentration of chlorogenic acid in the samples after heating was determined by ultraviolet spectrophotometry, and the retention rate was calculated.

#### 2.5.2. Light Stability Test

Chlorogenic acid samples and standard products with the same content were prepared into test solutions with the same concentration with deionized water, and each was divided into two groups: one group was placed on the laboratory windowsill for normal light exposure, and the other group was wrapped with light-tight tin foil for storage in the dark. On the 1st, 2nd, 3rd, 4th, 5th, 6th, 7th, 8th, 9th and 10th days, 50 μL of the sample solution was pipetted and mixed with 150 μL of DPPH ethanol solution with a concentration of 2 × 10^−5^ g/mL. After a dark reaction for 1 h, the absorbance at 517 nm was determined to calculate the DPPH free radical scavenging rate of each sample solution. The effects of light and dark treatment on chlorogenic acid solution and samples under different time gradients were compared.

#### 2.5.3. pH Stability Test

Chlorogenic acid samples and standard products with the same content were prepared into test solutions with the same concentration with deionized water, and each was divided into ten groups. The pH of the test solutions was adjusted to 1, 2, 3, 4, 5, 6, 7, 8, 9 and 10 respectively, and the solutions were standing at room temperature for 24 h. Then 50 μL of the sample solution was pipetted and mixed with 150 μL of DPPH ethanol solution with a concentration of 2 × 10^−5^ g/mL. After a dark reaction for 1 h, the absorbance of the sample solution at 517 nm was determined, and the free radical scavenging rate was calculated according to Formula (5) [42].

(6) $$Scavenging\;rate\;(\%)=\left(1-\frac{A_{sample}-A_{blank}}{A_{control}}\right)\times 100\%$$

where $A_{sample}$ is the absorbance after adding DPPH reagent to the sample; $A_{blank}$ is the absorbance of the pure sample; $A_{control}$ is the absorbance of the DPPH reagent. Thus, the effects of different pH conditions on chlorogenic acid solution and samples were compared. The higher the scavenging rate, the higher the content of chlorogenic acid under this condition, that is, the higher the stability. Each experiment was repeated three times.

### 2.6. Study on Chlorogenic Acid Antioxidant Activity

#### 2.6.1. Study on DPPH Free Radical Scavenging Capacity of CGA-SA-CMC

The DPPH free radical scavenging capacity of CGA-SA-CMC was determined according to the previous research method. Different concentrations of CGA-SA-CMC and CGA solutions were pipetted respectively and added to DPPH ethanol solution. After shaking well, the reaction was carried out at room temperature for 30 min, and the absorbance at 517 nm was determined. The DPPH free radical scavenging rate of each sample solution was calculated.

#### 2.6.2. Study on Fe^3+^ Reducing Capacity of CGA-SA-CMC

The potassium ferricyanide reduction method was referred to [43]: 1.0 mL of sample (CGA, CGA-SA-CMC) was taken, and 1 mL of 0.01 g/mL potassium ferricyanide was added in sequence. After shaking well, the reaction was carried out in a constant temperature water bath at 50 °C for 20 min, then quickly cooled with ice water, and 2.5 mL of 10% trichloroacetic acid was added. After mixing well, centrifugation was carried out at 4000 r/min with a high-speed centrifuge for 10 min. After centrifugation, the supernatant was taken and 0.5 mL of 0.1% ferric chloride solution was added. After mixing well and standing for 10 min, the absorbance was detected at 700 nm. The experiment was repeated three times and the average value was taken. The higher the absorbance value, the stronger the reducing capacity of the substance, and vice versa.

### 2.7. Cytotoxicity Determination

The biological safety of CGA-SA-CMC hydrogels can be tested by in vitro cell activity analysis with MTT method. First, the freeze-dried hydrogels were sterilized. Then, according to the ISO10993-5 standard [44], the hydrogel was extracted with normal saline to prepare extracts with different concentrations (0, 20 μg/mL, 40 μg/mL, 60 μg/mL, 80 μg/mL, 100 μg/mL) for testing the cytotoxicity of the extracts. 10^4^ fibroblasts (L929) were placed in each well of a 96-well plate and cultured for 24 h (5% CO_2_, 98% humidity, 37 °C). The same cells were divided into two groups: (1) negative control (L929 cells) and (2) positive control (gel bead extract added to the medium), and incubated for 24 h and 48 h. After the scheduled time, 20 μL of 5 mg/mL MTT was added to each well. After continuous incubation for 4 h, all the liquid was carefully taken out from the wells, and 150 μL of dimethyl sulfoxide was added. Shake for 15 s to ensure that the precipitate is completely dissolved. Finally, the absorbance at 490 nm was determined with a microplate reader (Allsheng, Feyond-A500, Hangzhou, China). The cell viability was calculated according to Formula (6).

(7) $$\mathrm{Cell}\;\mathrm{viability}\;(\%)=\frac{A_{sample}}{A_{control}}\times 100\%$$

where $A_{sample}$ is the absorbance of the experimental group; $A_{control}$ is the absorbance of the negative control group.

### 2.8. Statistical Analysis

Multiple group comparisons were performed using analysis of variance (ANOVA) combined with LSD post hoc test (IBM SPSS Statistics 27), with a significance level of *p* < 0.05. Experiments were repeated three times.

## 3. Results and Discussion

### 3.1. Structural Characterization of Bilayer SA/CMC Hydrogels

**Figure 2:**
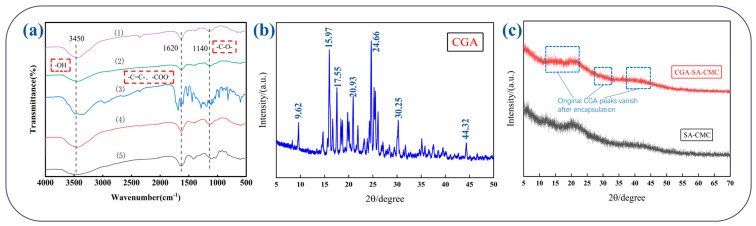
FTIR and XRD patterns of the sample: (**a**) FTIR spectra of (1) CGA-SA-CMC, (2) SA-CMC, (3) CGA, (4) CMC, and (5) SA, (**b**) X-ray diffraction pattern of CGA, (**c**) X-ray diffraction pattern of CGA-SA-CMC and SA-CMC.

The structural features of the hydrogels were characterized by FTIR and SEM to evaluate chemical interactions and surface morphology. FTIR spectra of CGA-SA-CMC, CGA-SA, and the starting materials are presented in Figure 2a. All samples display a broad peak near 3450 cm^−1^ corresponding to O–H stretching vibrations, a peak around 1620 cm^−1^ attributed to symmetric and asymmetric vibrations of carboxylate groups, and a peak near 1140 cm^−1^ associated with C–H bending and C–C stretching vibrations. The ester C–O stretching of chlorogenic acid also contributes to the absorption in this region. No new absorption peaks are observed in the spectra of the hydrogels compared with the pure components, indicating that no chemical reaction occurred during bead formation, and the encapsulation of CGA is primarily through physical entrapment.

The sharp diffraction peaks in the XRD pattern of CGA indicate long-range order in its molecular arrangement, corresponding to a typical crystalline structure (Figure 2b). In contrast, the diffraction peaks of the loaded CGA-SA-CMC completely disappear, suggesting that chlorogenic acid has been transformed into an amorphous state (Figure 2c). This state implies that CGA molecules are highly dispersed and no longer aggregate to form crystals. In drug or active ingredient delivery systems, such a transformation is generally beneficial: the amorphous state is a high-energy state with high surface free energy, and usually exhibits higher apparent solubility and faster dissolution rate than the crystalline state, which helps improve bioavailability.

**Figure 3:**
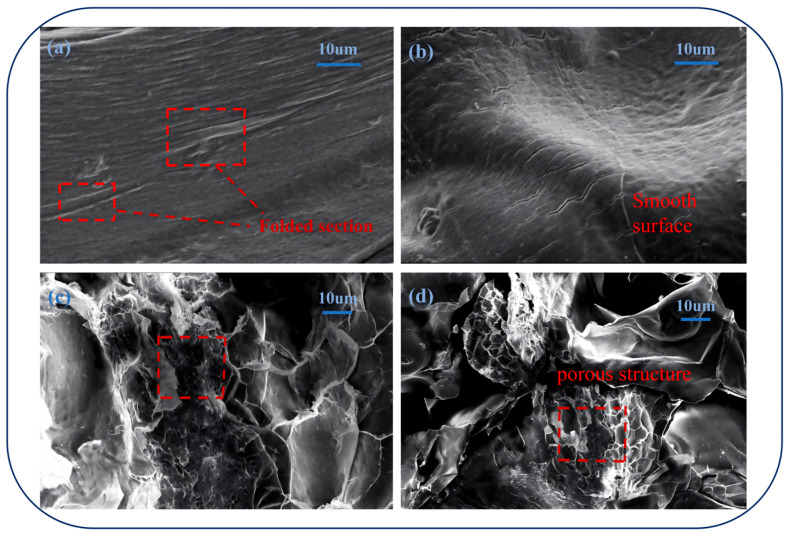
SEM images of freeze-dried hydrogels: CGA-SA (**a**) ×1500; CGA-SA-CMC (**b**) ×1500; (**c**,**d**): CGA-SA-CMC cross-section (×1000).

SEM images of the freeze-dried hydrogels reveal distinct differences in surface morphology (Figure 3). CGA-SA hydrogels exhibit wrinkles and fibrous textures, reflecting a relatively loose and heterogeneous network. After CMC coating, CGA-SA-CMC hydrogels show a markedly smoother and denser surface, with the previous wrinkles and fibrous features largely eliminated. The denser surface structure suggests that the CMC layer effectively fills surface irregularities, enhancing encapsulation efficiency, reducing premature CGA leakage, and improving mechanical stability. Together, the FTIR, XRD and SEM results confirm that the bilayer hydrogels maintain physical integrity without chemical modification, and the CMC coating strengthens the structural robustness and surface uniformity, while the amorphous state of CGA in the gel contributes to improved bioavailability, supporting their potential for controlled gastrointestinal delivery.

### 3.2. Swelling Behavior and pH-Responsive Performance

**Figure 4:**
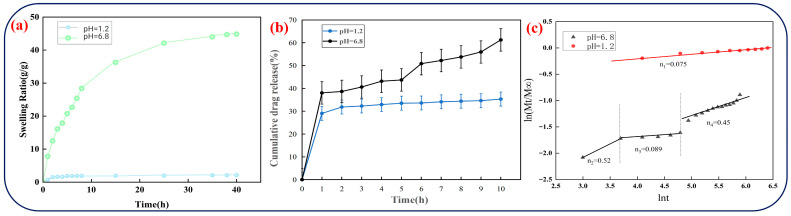
Swelling behavior, cumulative release, and release kinetics of CGA from CGA-SA-CMC hydrogels under simulated gastric (pH 1.2) and intestinal (pH 6.8) conditions: (**a**) swelling curves, (**b**) cumulative release profiles, (**c**) Korsmeyer-Peppas model fitting for release mechanism.

The swelling behavior of CGA-SA-CMC hydrogels was investigated under different pH conditions to evaluate their pH-responsive characteristics (Figure 4a). The hydrogels can absorb a large amount of water, with a maximum swelling ratio of 44.17 g/g. This is attributed to the abundant porous network structure within the gel formed by SA/CMC calcium ion cross-linking (Figure 3c,d), consistent with existing studies on the internal structural characteristics of SA/CMC calcium ion gels [45,46]. This porous network allows water to penetrate efficiently, facilitating material exchange and providing space for drug loading. A higher swelling ratio enables the hydrogel to accommodate more drug molecules, while also improving diffusion channels and avoiding entrapment of CGA within the network.

**Figure 5:**
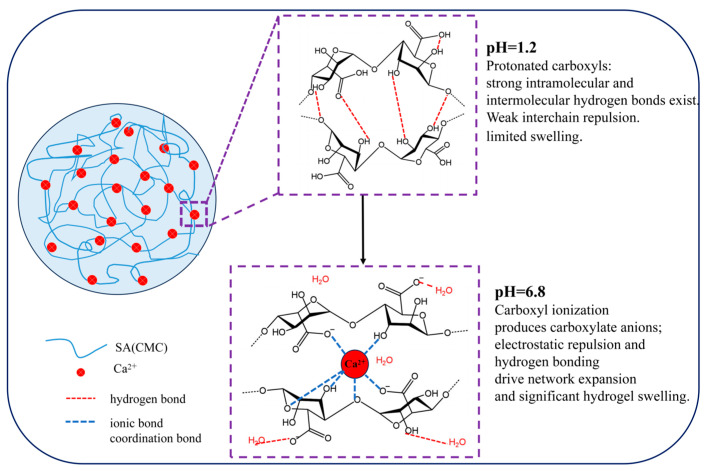
Schematic of pH-responsive swelling mechanism.

The swelling ratios exhibit clear pH dependence. At pH 1.2, most carboxyl groups on the sodium alginate chains exist in a protonated form, and hydrogen bonding dominates, resulting in minimal network expansion and limited swelling. In contrast, at pH 6.8, carboxyl groups ionize to form carboxylate anions, and electrostatic repulsion among anions, combined with hydrogen bonding with water molecules, drives network expansion, allowing the hydrogels to swell significantly (Figure 5). This pH-responsive swelling behavior indicates that the hydrogel can remain compact in acidic gastric conditions while expanding in intestinal conditions, which is beneficial for controlled CGA release.

The total drug loading capacity of the hydrogels was determined to be 15.2–16.7%, The encapsulation efficiency of CGA ranges from 65.5% to 67.1%, confirming that the hydrogels can effectively encapsulate CGA. In traditional liposome systems, the drug loading capacity and encapsulation efficiency of CGA reached 8.70 ± 0.23% and 51.34 ± 0.32%, respectively. After TPGS modification, these parameters increased to 11.21 ± 0.02% and 83.22 ± 0.11% [25]. This indicates that the gel system demonstrates superior drug loading capacity and encapsulation efficiency compared to conventional liposomes, although its encapsulation efficiency still lags behind that of specially modified liposomes. In vitro release studies under simulated gastrointestinal conditions further demonstrated pH-dependent release (Figure 4b). Univariate analysis of variance revealed that pH conditions had a highly significant effect on the cumulative drug release amount of samples (*p* < 0.05). Post hoc LSD testing indicated that at all time points from 1 to 10 h, the cumulative drug release amount under pH = 6.8 conditions was significantly higher than that under pH = 1.2 conditions (*p* < 0.05). In simulated gastric fluid (pH 1.2), CGA release reached only 29.05% within 1 h and plateaued at ~30%, consistent with the low swelling at this pH. Under simulated intestinal fluid (pH 6.8), the release reached 38.03% at 1 h and gradually increased to nearly 70% at 10 h, in agreement with the enhanced swelling behavior. In studies on encapsulating chlorogenic acid using liposomal systems, drug release reached nearly 80% within 4 h under gastric conditions. Conversely, the drug release in intestinal environments was significantly lower than that in gastric conditions. This reported system also demonstrated controlled release of CGA, but the excessively rapid drug release in gastric environments was detrimental to the utilization of CGA [25]. Excessive CGA release from the gastric cavity is also observed in starch-gel complex encapsulation systems [47]. Compared to these systems, this study demonstrates certain advantages in controlled release within the gastrointestinal tract. pH 1.2 and 6.8 were selected as representative gastric and intestinal conditions, respectively, which are commonly used benchmark environments in in vitro gastrointestinal simulation studies for pH-responsive drug delivery systems [33,34,38]. Intermediate pH values (4.5–5.5) correspond to a transitional physiological state and are expected to exhibit a continuous swelling-mediated release behavior of the hydrogel system rather than a distinct release profile. During this process, the gel mass remained nearly unchanged, indicating negligible degradation under the tested gastrointestinal conditions, consistent with previous studies on similar polysaccharide-based hydrogel systems [48,49].

Drug release kinetics were analyzed using the Korsmeyer–Peppas model (Figure 4c). At pH 1.2, the release exponent n_1_ < 0.43, indicating a Fickian diffusion-controlled mechanism dominated by concentration-driven molecular diffusion. Under pH 6.8 conditions, the release behavior exhibited a distinct stage-dependent profile that could be divided into three consecutive phases, with diffusion exponents n2, n3, and n4 of 0.52, 0.089, and 0.45, respectively. The intermediate stage (n_3_ = 0.089, 40.4–121.5 min) corresponds to a diffusion-dominated regime, suggesting a transient period of Fickian transport within a relatively stable hydrogel network. The initial (n_2_ = 0.52, t < 40.4 min) and late stages (n_4_ = 0.45, t > 121.5 min) fall within the anomalous transport range, indicating a coupled mechanism governed by both Fickian diffusion and polymer chain relaxation associated with hydrogel swelling. Overall, the release behavior can be described by a diffusion-swelling coupling mechanism, in which hydrogel swelling progressively modulates mass transport pathways and diffusional resistance over time. The bilayer hydrogel structure further regulates this process, suppressing premature gastric release and enabling sustained intestinal delivery.

### 3.3. Stability of Chlorogenic Acid in CGA-SA-CMC Hydrogels

**Figure 6:**
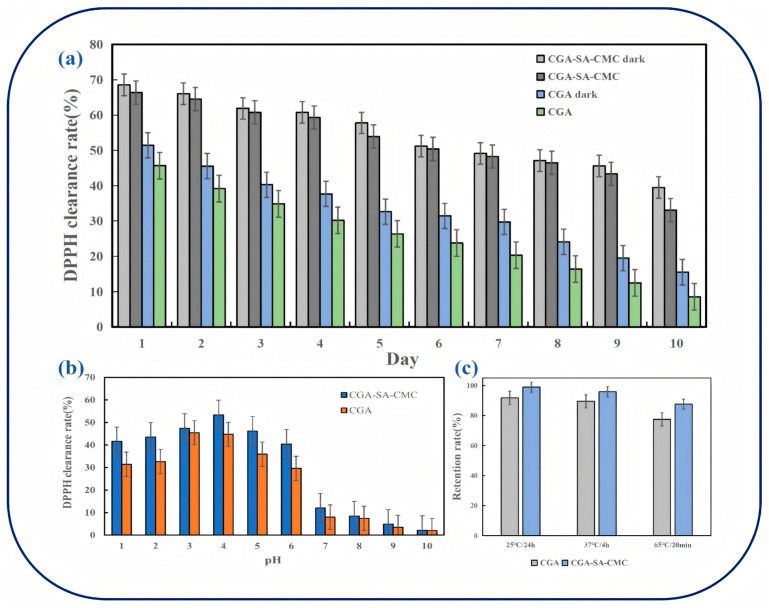
Thermal, light, and pH stability of CGA in CGA-SA-CMC hydrogels compared with free CGA, assessed by retention and DPPH scavenging activity: (**a**) DPPH radical scavenging rates of CGA and CGA-SA-CMC under light and dark conditions, (**b**) DPPH radical scavenging rates of CGA and CGA-SA-CMC under various pH conditions, (**c**) Retention rate of chlorogenic acid in CGA and CGA-SA-CMC after treatment at varying temperatures for specified durations.

The stability of CGA encapsulated in CGA-SA-CMC hydrogels was evaluated under light, pH, and thermal conditions (Figure 6).

**Light Stability**: As shown in Figure 6a, CGA-SA-CMC hydrogels exhibited higher resistance to photodegradation compared with CGA solution. Analysis of variance revealed significant differences in sample type, light treatment, and time (*p* < 0.05). Pairwise comparisons indicated that the CGA-SA-CMC light-shielding group exhibited the highest activity and optimal stability. Light exposure significantly reduced the clearance rate, but modified embedding effectively mitigated photodegradation. Pure CGA suffered the most severe degradation under light exposure. All intergroup differences reached statistical significance (*p* < 0.05). Under continuous light exposure over 10 days, the DPPH radical scavenging rate of CGA-SA-CMC decreased from 58.27% to 38.92%, a reduction of 33.35%, whereas CGA solution decreased from 60.53% to 38.17%, a reduction of 37.12%. Under dark conditions, the decrease was smaller: 29.03% for CGA-SA-CMC versus 35.92% for CGA solution. These results indicate that hydrogel encapsulation can moderately protect CGA from light-induced degradation, consistent with previous reports highlighting CGA’s sensitivity to light [8,19].

**pH Stability**: The pH-dependent stability of CGA was evaluated by DPPH scavenging activity (Figure 6b). Both CGA solution and CGA-SA-CMC hydrogels reached maximum activity at pH 4, with values of 45.2% and 49.3%, respectively. As pH increased, activity gradually decreased. Notably, at pH 7, the DPPH clearance of CGA-SA-CMC hydrogels remained at 37.8%, compared with 31.5% for CGA solution, indicating the hydrogel matrix helps maintain CGA activity under mild alkaline conditions. Under strongly alkaline conditions (pH 9–10), the protective effect was reduced, and both samples showed similar lower activity (~12–15%). Statistical analysis revealed that within the pH range of 1 to 9, the free radical scavenging rate of CGA-SA-CMC was significantly higher than that of CGA (*p* < 0.05). Under pH 10 conditions, the free radical scavenging rate of CGA-SA-CMC remained significantly higher than that of CGA (*p* < 0.05). Overall, these results indicate that CGA-SA-CMC exhibits significantly superior stability compared to CGA under varying pH conditions. This aligns with the pH-responsive behavior of the hydrogel, which swells more in neutral conditions, thereby moderating CGA release and protecting its stability.

**Thermal Stability**: CGA retention after thermal treatment is shown in Figure 6c. After 24 h at 25 °C and 4 h at 37 °C, retention in hydrogels remained above 90%, while CGA solution showed slightly lower retention (~88% at 25 °C, ~85% at 37 °C). At 65 °C for 20 min, retention decreased to over 80% in hydrogels, compared with ~75% in CGA solution. The differences are modest, suggesting that encapsulation provides limited protection against thermal degradation. However, under all heat treatment conditions, the activity retention rate of CGA-SA-CMC was significantly higher than that of CGA (*p* < 0.05), indicating that SA-CMC encapsulation can enhance the thermal stability of CGA to some extent.

In summary, the bilayer SA/CMC hydrogel matrix can moderately enhance CGA stability, particularly under light exposure and mild alkaline conditions, while thermal protection is limited. The gel system demonstrates consistent efficacy in enhancing CGA stability when applied to other polyphenolic compounds such as anthocyanins [37,38]. These results, combined with the pH-responsive swelling behavior, indicate that the hydrogel is suitable for gastrointestinal delivery of CGA, offering protection during storage and controlled release in the intestine.

### 3.4. Antioxidant Activity and Biocompatibility of CGA-SA-CMC Hydrogels

**Figure 7:**
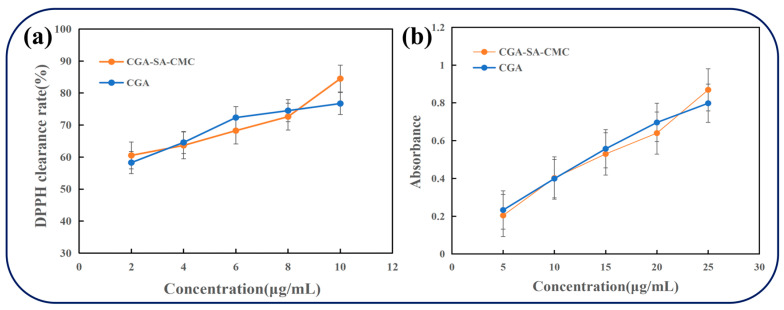
Antioxidant activity of CGA solution and CGA-SA-CMC hydrogels at varying concentrations: (**a**) DPPH radical scavenging, (**b**) Fe^3+^ reducing capacity.

The in vitro antioxidant activity of chlorogenic acid (CGA) encapsulated in SA/CMC hydrogels was evaluated using DPPH radical scavenging and Fe^3+^ reducing assays (Figure 7). The comprehensive antioxidant experiment results demonstrated that concentration significantly influenced the difference in antioxidant activity between the two compounds (*p* < 0.05). At medium and low concentrations, there was no significant difference in antioxidant activity between CGA and CGA-SA-CMC (*p* > 0.05). However, at higher concentrations, the antioxidant activity of CGA-SA-CMC was significantly higher than that of CGA (*p* > 0.05). The DPPH scavenging capacity of both CGA solution and CGA-loaded hydrogels increased with CGA concentration (2–10 μg/mL), demonstrating a clear dose-dependent effect (Figure 7a). At each concentration, the scavenging rates of the hydrogels were comparable to those of free CGA: for example, at 6 μg/mL, the DPPH scavenging was 68.26% for CGA solution and 72.34% for hydrogels; at 10 μg/mL, 84.51% for solution and 76.73% for hydrogels. The slightly lower activity of hydrogels at higher concentrations likely reflects the sustained-release effect, where CGA is gradually released from the hydrogel network, resulting in prolonged radical scavenging. In the CGA delivery system derived from flaxseed oligosaccharides conjugates at a concentration of 1 mg/mL, the DPPH free radical scavenging efficiency reached 63.74 ± 1.46% [50], whereas the gel system exhibited higher retention of free radical scavenging capacity. Overall, the hydrogels maintained the inherent antioxidant activity of CGA while enabling controlled release.

The reducing power assay further confirmed the antioxidant capacity of CGA hydrogels. The absorbance at 700 nm, which correlates with Fe^3+^ reduction, increased with CGA concentration for both the solution and hydrogel samples (Figure 7b). At equivalent CGA concentrations, the hydrogels exhibited similar reducing capacity to free CGA, indicating that encapsulation did not compromise electron-donating ability. The rapid swelling of hydrogels under assay conditions facilitated the release of CGA, allowing efficient interaction with Fe^3+^ ions. In liposome systems, the in vivo antioxidant activity of CGA was moderately enhanced, but its antioxidant activity under more conditions has not been reported [25,51].

**Figure 8:**
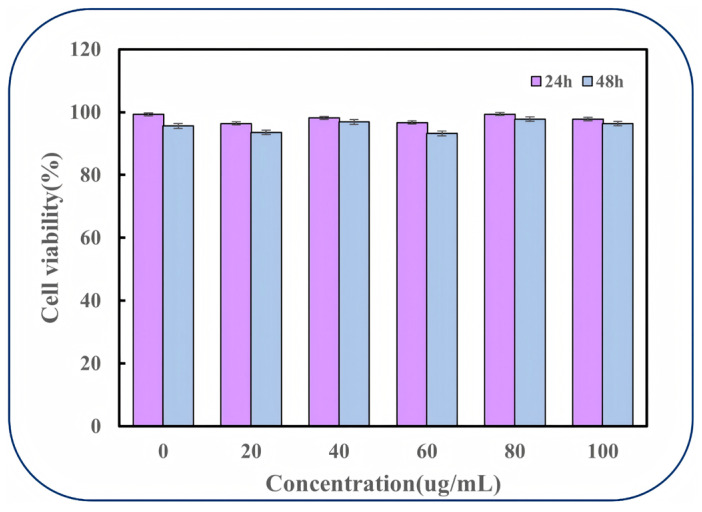
Cell viability of L929 fibroblasts after 24 h and 48 h exposure to CGA-SA-CMC hydrogel extracts at varying concentrations.

The biocompatibility of CGA-SA-CMC hydrogels was evaluated using the MTT assay on L929 cells. Within the experimental concentration range of 0–100, the relative cell retention rate of CGA-SA-CMC remained above 89% after incubation for 24 h and 48 h, respectively. Univariate analysis of variance and multiple comparison tests revealed no significant differences among concentration groups compared to the blank control group (*p* > 0.05). Furthermore, prolonged incubation to 48 h maintained high cell viability levels, indicating that CGA-SA-CMC exhibited no significant cytotoxicity. As shown in Figure 8, the cell survival rates after 24 h and 48 h incubation with hydrogel extracts at six different concentrations all exceeded 95%, indicating negligible cytotoxicity. These results demonstrate that the prepared CGA-SA-CMC hydrogel is non-toxic and possesses certain biocompatibility. This experiment also has certain limitations. It should be noted that further in vivo studies or investigations using human gastrointestinal cells are required to thoroughly validate the in vivo efficacy of this hydrogel, thereby providing stronger evidence to support its potential applications in biomedical or food-related delivery systems.

## 4. Conclusions

In summary, this study developed a novel CGA delivery system based on SA-CMC hydrogel. As a carrier, this hydrogel enhances the stability of CGA. In vitro results indicate that this pH-responsive sustained-release system improves the dissolution profile of chlorogenic acid, demonstrating potential for enhancing the bioavailability of CGA. In vitro release studies demonstrated that CGA release is triggered by pH conditions: approximately 30% is released under simulated gastric conditions, while sustained release occurs under simulated intestinal conditions, reaching about 70% at 10 h. Compared with free CGA, the thermal, light, and pH stability of CGA were significantly improved in the hydrogel system, and its intrinsic antioxidant activity was largely preserved. These findings indicate that the CGA-SA-CMC delivery system effectively enhances the stability of chlorogenic acid and may potentially improve its bioavailability, supporting its potential for broader applications of CGA.

Looking forward, further studies are warranted to evaluate in vivo pharmacokinetics, long-term storage stability, and the potential effects of hydrogel composition on release kinetics. Further studies may focus on comprehensive in vivo evaluation of this hydrogel system to better support its potential applications in biomedical and food-related delivery fields. More systematic biological investigations under physiological conditions could also provide additional insights into its functional performance. Although this study focused on a specific hydrogel formulation and in vitro assays, these limitations do not compromise the novelty of the system, and the findings provide a solid foundation for the rational design of CGA delivery platforms for food, nutraceutical, and biomedical applications. Simultaneously, this hydrogel system has potential for further development into practical oral delivery formulations, such as capsules, paste-like gels, or powder-based formulations, enabling its application in functional foods, oral supplementation, and related antioxidant delivery scenarios.

## Data Availability

The original contributions presented in this study are included in the article. Further inquiries can be directed to the corresponding authors.
